# A decisive bridge between innate immunity and the pathognomonic morphological characteristics of type 1 diabetes demonstrated by instillation of heat-inactivated bacteria in the pancreatic duct of rats

**DOI:** 10.1007/s00592-022-01881-4

**Published:** 2022-04-24

**Authors:** Tegehall Angie, Ingvast Sofie, Melhus Åsa, Skog Oskar, Korsgren Olle

**Affiliations:** 1 Department of Immunology, Genetics and Pathology, Rudbeck Laboratory, Uppsala University, 751 85 Uppsala, Sweden; 2 Department of Medical Sciences, Section of Clinical Microbiology, Uppsala University, Uppsala, Sweden

**Keywords:** Type 1 Diabetes, Innate immunity, Acquired immunity, Insulitis, Bacteria, Animal model

## Abstract

**Aims:**

Periductal inflammation and accumulation of granulocytes and monocytes in the periislet area and in the exocrine pancreas is observed within hours after instillation of heat-inactivated bacteria in the ductal compartment of the pancreas in healthy rats. The present investigation was undertaken to study how the acute inflammation developed over time.

**Methods:**

Immunohistochemical evaluation of the immune response triggered by instillation of heat-inactivated bacteria in the ductal compartment in rats.

**Results:**

After three weeks, the triggered inflammation had vanished and pancreases showed normal morphology. However, a distinct accumulation of both CD4+ and CD8+ T cells within and adjacent to affected islets was found in one-third of the rats instilled with heat-inactivated *E. faecalis*, mimicking the insulitis seen at onset of human T1D. As in T1D, this insulitis affected a minority of islets and only certain lobes of the pancreases. Notably, a fraction of the T cells expressed the CD103 antigen, mirroring the recently reported presence of tissue resident memory T cells in the insulitis in humans with recent onset T1D.

**Conclusions:**

The results presented unravel a previously unknown interplay between innate and acquired immunity in the formation of immunopathological events indistinguishable from those described in humans with recent onset T1D.

## Introduction

The autoimmune barrier in Type 1 Diabetes (T1D) is only vaguely understood, and the clinical relevance of available rodent models is debated [1–4]. However, especially the NOD mouse model has fertilized investigations on autoimmunity in T1D, despite the seemingly low predictive value of this model for clinical intervention therapies [1, 3, 5–8]. An immunopathological hallmark in the pancreas of patients with T1D is the accumulation of immune cells within and around affected islets, i.e., insulitis [9, 10]. Notably, only few T cells infiltrate the islets [3, 10, 11]. In fact, an extensive study using multiplex immunofluorescent staining of 35 simultaneous biomarkers with a spatial resolution of 1 µm demonstrated that immune and islet cells essentially remain isolated from each other even in patients with recent onset T1D [12]. Phenotypically, CD8^+^ and CD4^+^ T cells dominate the insulitis followed by monocytes/macrophages. B cells are less frequent, whereas regulatory T cells, NK cells and plasma cells are only rarely found [13]. Also, the exocrine pancreas is affected, as evidenced for example by a markedly smaller volume and findings of multifocal T-cell infiltrates in acinar regions [9]. The proportion of islets with insulitis has been inversely associated with disease duration in some [9], but not other studies [10]. Insulitis seems not related to age at onset, number of autoantibodies, or HLA genotype [9]. The presence of remaining insulin-positive cells in islets with insulitis several years, or even decades, after diagnosis of T1D is intriguing and suggests a mild and slowly progressing disease.

A substantial proportion the T cells in the insulitic lesions from subjects with recent onset T1D constitute tissue resident memory T cells (T_RM_ cells) [14]. T_RM_ cells were present in all T1D subjects examined and represented about 40% of the total number of T cells per inflamed islet, a proportion of T_RM_ cells similar to that previously described in skin lesions of psoriasis [15]. Also, T and B cell gene expression pattern in infiltrated islets argue against that the T cells found in the insulitic lesions constitute conventional cytotoxic CD8+ T cells [11]. Preproinsulin (PPI) is considered a major T cell recognized autoantigen in T1D; however, in a recent study a total number of only 24 PPI_15–24_ reactive CD8+ T cells were found when examining 357 islets in 4 subjects with recent onset T1D [16].

T_RM_ cells constitute a subset of memory T cells that persist for years at the site of a previous infection without persistence of antigen stimulation and provide rapid immune protection against re-infection via the same entry port [17]. The substantial proportion of T_RM_ cells in islets of recent onset T1D subjects supports the hypothesis of infectious agents in the development of T1D [18].

We have previously reported that injection of heat-inactivated human pathogens in the ductal compartment of the pancreas in healthy rats of several strains causes periductal inflammation and accumulation of immune cells, mainly granulocytes and monocytes, in the exocrine pancreas and in the peri-islet area [19]. Small bleedings or large dilatations of the capillaries were frequently found within the islets, and several beta cells showed severe hydropic degeneration, i.e., swollen cytoplasm, but with preserved nuclei. These findings show marked similarities with those observed in the pancreases of patients dying at onset of T1D [20]. However, inflammation of the pancreas in subjects examined weeks or months after diagnosis of T1D is substantially reduced and mainly consists of discreet insulitis, mainly consisting of T cells, affecting a minority of islets [11, 21].

The present investigation was undertaken in order to study how the acute inflammation triggered by instillation of heat-inactivated bacteria in the ductal compartment develops over a period of several weeks with an aim to investigate a potential association of innate immunity to the formation of insulitis.

## Materials and methods

### Human pancreatic samples

Pancreatic tissue from a 29-year-old organ donor that died at onset of T1D (a previously healthy man with B-glucose 46 mmol/L and ketoacidosis at arrival to the emergency room and with a BMI of 24.2 kg/m^2^ and HbA1c 90 mmol/mol, previously described in detail [19] and two donors without diabetes (a 31-year-old woman with BMI 25.4 kg/m^2^ and HbA1c 33 mmol/mol, and a 27-year-old man with BMI 26.0 kg/m^2^ and HbA1c 39 mmol/mol), procured within the Nordic Network for Clinical Islet Transplantation, were included in the study. Biopsies were formalin-fixed and paraffin-embedded or frozen in liquid nitrogen and stored at − 80 °C.

### Bacteria

All four strains used in our earlier study were included, i.e., *α*-hemolytic streptococci, Escherichia coli, Enterococcus faecalis and Staphylococcus aureus (6). They were isolated from patients with invasive infections at the Department of Clinical Microbiology, Uppsala University Hospital, Uppsala, Sweden, and chosen for their documented ability to translocate into pancreas and cause infections in this anatomic region [22–25]. The bacteria were grown overnight in brain heart infusion (BHI) broth (Becton Dickinson) or Trypticase soy broth BBL with 10% inactivated horse serum and 5% Fildes enrichment BBL at 35 ℃ to a concentration of 10^9^ CFU/mL. After heat-inactivation by boiling for 15 min, the viability was controlled. The dead bacteria were stored at − 70 °C until used.

### Animals and operating procedure

Fifty-three healthy male Wistar rats weighing 250 to 300 g (Taconic, Denmark) were used. Before the bacterial challenge, the animals were kept under standard laboratory conditions in accordance with the National Institute of Health principles of laboratory animal care and national laws in Sweden. The rats were housed two by two in plastic cages under a 12:12-h light–dark cycle, and they were given water and food ad libitum. At challenge, 200 µl of BHI broth with or without bacteria was instilled as previously described [19]. Animals were subsequently kept under normal conditions for 4 h, 3 or 6 weeks, respectively. At the end of the experiment after 3 or 6 weeks, glucose tolerance was evaluated on some of the rats by an intravenous glucose tolerance test IVGTT under full anesthesia (thiobutabarbital sodium administered 10 min before glucose injection (100 mg/kg BW intraperitoneally). Bolus injection of glucose was given within 60 s via the tail vein. Blood glucose was measured immediately before and 5, 10, 30, 60, 90 and 120 min after glucose injection. Blood glucose was measured with a glucometer (CONTOUR^®^, Bayer, Solna, Sweden), operating within a range of 0.6–33.3 mmol glucose/L.

Animals were subsequently killed by heart puncture. Serum, plasma and pancreas were collected. The head and tail of the pancreas were fixed in 4% paraformaldehyde and prepared for paraffin embedding.

### RNA extraction and qPCR array

Frozen tissue biopsies from three different parts of the pancreatic body and tail from the organ donor with recent onset T1D and one biopsy from each of the two non-diabetic organ donors were subjected to sectioning and RNA extraction. Twenty consecutive 10 µm sections were placed on glass slides for subsequent IHC (Sects. 1–2, 7–8, 13–14, & 19–20) or placed in 600 µl buffer RLT (Qiagen) containing 1% 2-mercaptoethanol (Sigma-Aldrich) for extraction of RNA. From each of the five tissue biopsies, Sects. 3–6, 9–12 and 15–18 were pooled and RNA extracted separately. Frozen tissue from the head and the tail of the pancreas of four rats killed 4 h after the instillation of *Enterococcus faecalis* in the ductal system was subjected to sectioning and RNA extraction following the same protocol as for the human samples.

AllPrep Mini kit (Qiagen) was used for RNA extraction according to the manufacturer’s instructions, including homogenization using QiaShredder columns (Qiagen) and on-column DNase digestion. In the final step, an elution volume of 30 µL was used, giving RNA concentrations ranging from 63 to 217 ng/µL per sample.

Pathway-specific primer mixes (Rat Antibacterial Response, PBR-148Z, and Human Antibacterial Response, PBH-148Z; Qiagen) were used for preamplification, and qPCR arrays (Rat Antibacterial Response, PARN-148ZE, and Human Antibacterial Response, PAHS-148ZC; Qiagen) were used for the expression analysis of 84 genes involved in innate antibacterial responses in human and rat, respectively. Genes with a quantification cycle (Cq) value > 35 were regarded as non-detected and assigned a Cq of 35 to calculate fold induction.

### Immunohistochemistry

Formalin-fixed and paraffin-embedded pancreas biopsies were cut into 6 µm consecutive sections and processed for immunohistochemistry for paraffin sections, as previously described [19]. In brief, antigens were unmasked by heat-induced antigen retrieval, using buffer sodium citrate or EDTA according to the manufacturers’ recommendations. Synaptophysin and CD45 or insulin and CD3 double-staining was used for screening for insulitis within the human pancreases. Insulin and CD43 double-staining was used for screening for insulitis within the rat pancreases. Consecutive sections were further stained for CD3, CD4, CD8, CD20, CD68, CD103, insulin and glucagon (Table 1). Bound antibodies were visualized using Dako EnVision and diaminobenzidine-based substrate or double stained using EnVision G/2 Double Stain System, Rabbit/Mouse (DAB + /Permanent Red). Sections were counterstained with hematoxylin and analyzed by light microscopy Leica. Rat spleen sections were used as positive control for all antibodies. Negative controls had the primary antibody replaced by buffer.

**Table 1:** Detailed list of antibodies used

Name	Clone and supplier	HIER pH	Dilution
Anti-CD3 (rat)	SP7 (Abcam)	6.0	1:100
Anti-CD4 (rat)	CAL4 (Abcam)	9.0	1:4000
Anti-CD8 (rat)	OX-8 (Abcam)	6.0	1:1000
Anti-CD20 (rat)	SP32 (Abcam)	6.0	1:100
Anti-CD43 (rat)	AbD (BioRad)	6.0	1:200
Anti-CD68 (rat)	ED1 (Biorad)		1:100
Anti-CD103 (rat)	ERP2259027 (Abcam)	9.0	1:1000
Anti-insulin	A0564 (Agilent))		1:200
Anti-glucagon	LS C312053 (LSBIO)	6.0	1:400
Anti-CD45 (human)	2B11 + PD7/26 (Agilent)	9.0	1:75
Anti-synaptophysin	DAK-SYNAP (Agilent)	9.0	1:100
Anti-CD3	Polyclonal (Agilent)	9.0	1:100

### Statistical analyses

Data from the IVGTT are presented as means ± SEM. The statistical significance of the differences between groups was analyzed by the Kruskal–Wallis test followed by Dunn's test for multiple comparisons. PCR array data were analyzed with nonparametric testing using Qlucore Omics Explorer version 3.3 software with an interface to R (Qlucore, Lund, Sweden). FDR was determined using the Benjamini Hochberg procedure.

## Results

### Bacterial challenge and animal well-being

**Fig. 1:**
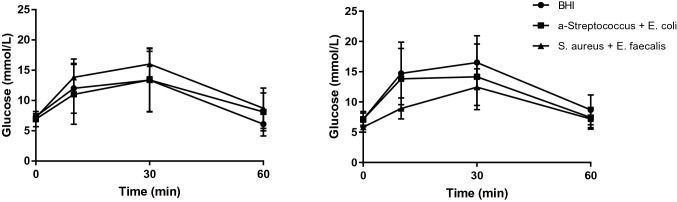
Intravenous glucose tolerance test revealed no significant differences in peak glucose values and subsequent glucose disposal 3 weeks (left panel) or 6 weeks (right panel) after bacterial challenge with *S. aureus* and *E. faecalis* (filled squares) or *E. coli* and *α*-hemolytic streptococcus (filled triangles) when compared with rats instilled with brain heart infusion broth alone (filled circles; *P* > 0.05)

All animals tolerated the surgical procedure well. No macroscopic changes were observed in any abdominal organ 3 or 6 weeks after the bacterial challenge. IVGTT revealed no significant differences in peak glucose values and subsequent glucose disposal 3 or 6 weeks after the bacterial challenge when compared with control rats (*P* > 0.05, Fig. 1).

### Early innate antibacterial responses

**Fig. 2:**
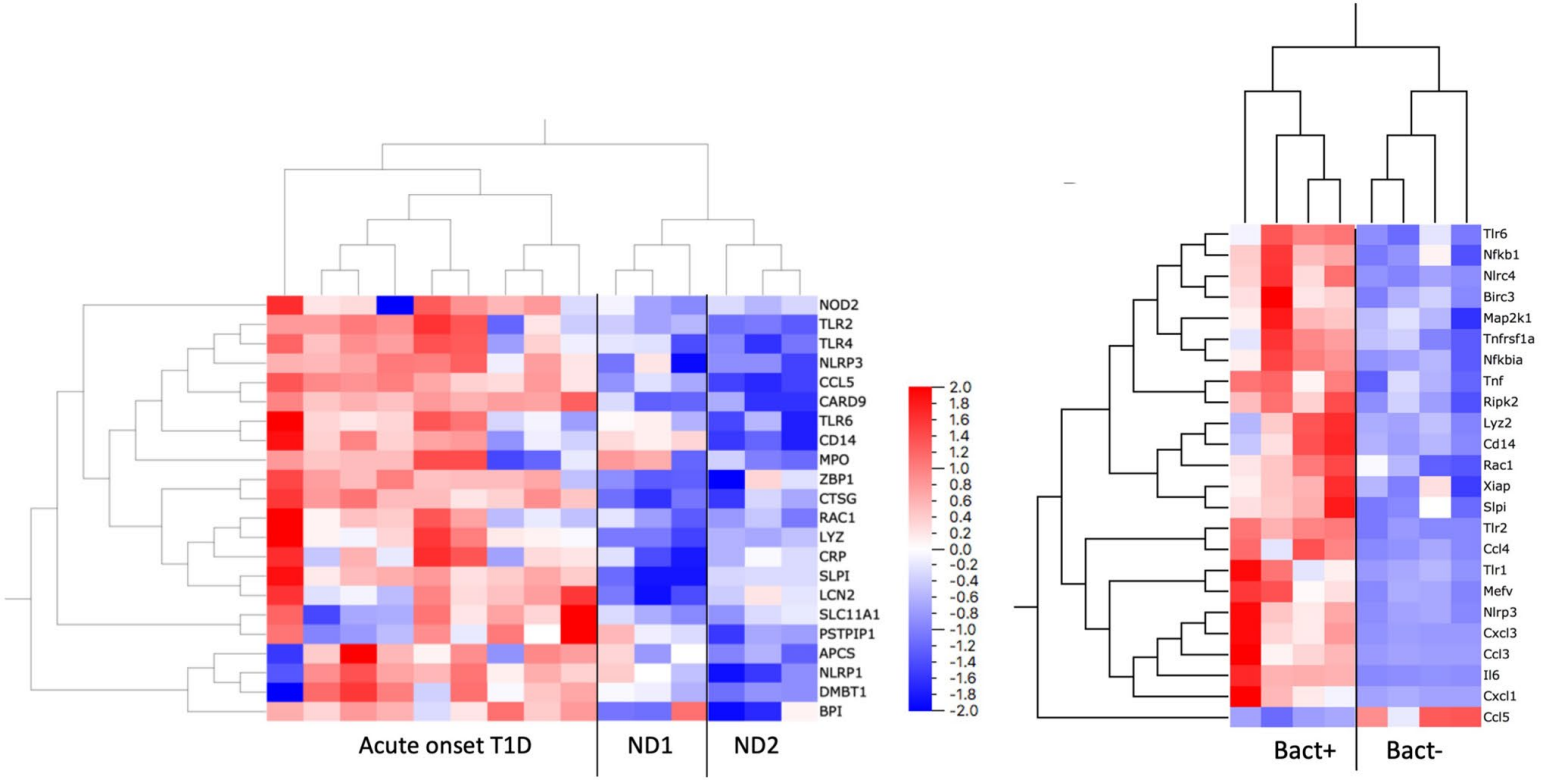
Expression of 84 human genes related to innate antibacterial response was analyzed with a qPCR array (left panel). Pancreatic RNA extracted from an organ donor that died at acute onset of T1D showed a > twofold overexpression of 18 antibacterial genes compared to non-diabetic donors (ND1 & ND2) (*P* < 0.05, fdr < 10%). Expression of 84 rat genes related to innate antibacterial response was analyzed with a qPCR array. RNA was extracted from pancreatic rat tissue with (bact +) or without (bact-) instilled bacteria (right panel). Bacterial translocation induced a > twofold overexpression of 22 genes and under expression of one gene (ccl5/rantes) related to antibacterial response (*P* < 0.05, fdr < 10%). Eight of these were homologues to genes that were significantly induced by bacterial translocation in the human tissue samples

Pancreatic sections from a human organ donor with acute onset T1D showed a > twofold overexpression of 18 antibacterial genes compared to non-diabetic donors (*P* < 0.05, fdr < 10%) (Fig. 2 left panel). Bacterial translocation in the rat induced after 4 h a > twofold overexpression of 22 genes and underexpression of one gene (ccl5/rantes) related to antibacterial response (*P* < 0.05, fdr < 10%) (Fig. 2 right panel). Eight of these where homologues to genes that were significantly induced by bacterial translocation in the human.

### Immune-cell infiltration and insulitis

#### A human organ donor that died at onset of type 1 diabetes

**Fig. 3:**
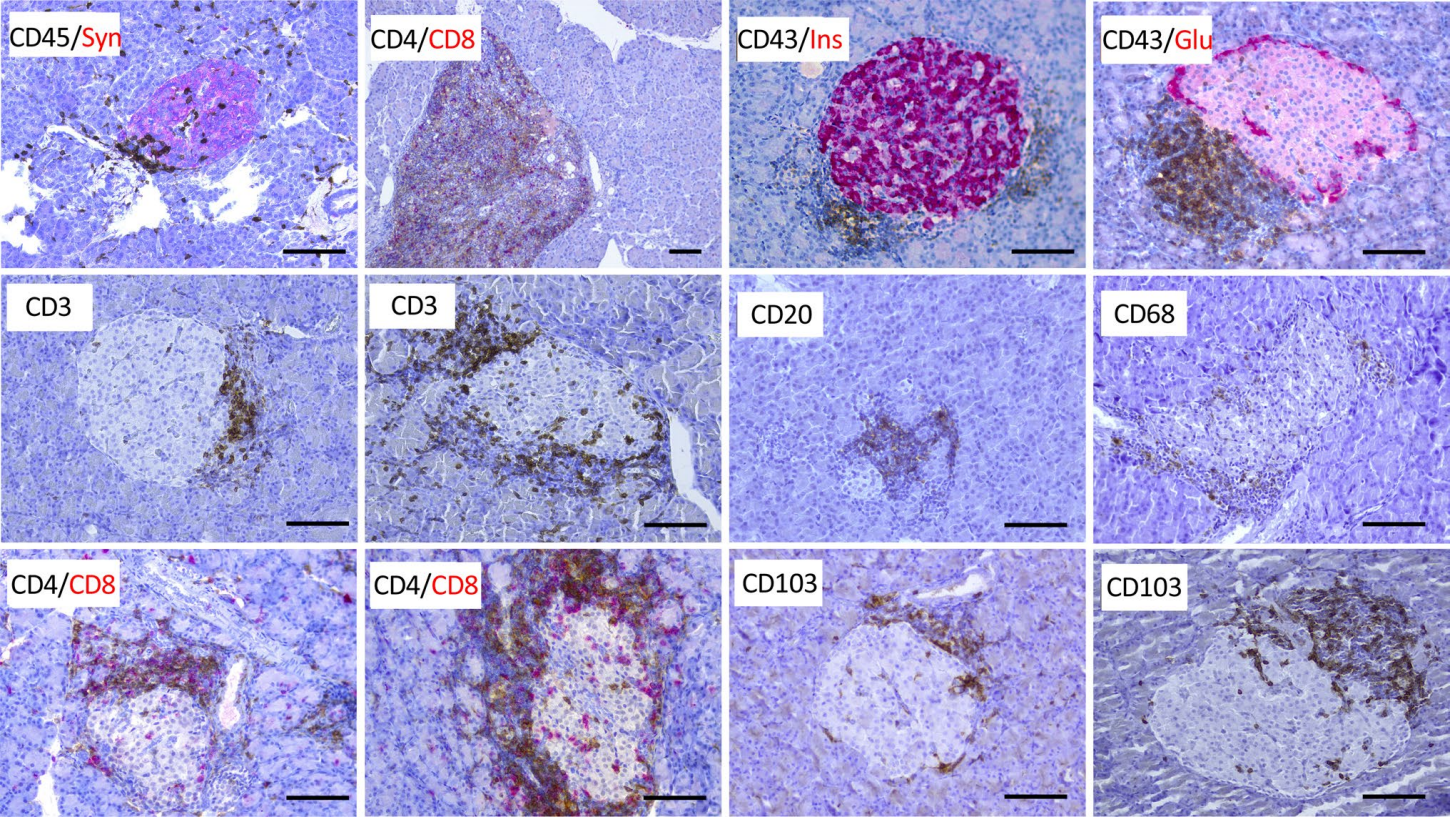
Pancreatic tissue from a 29-year-old organ donor that died at onset of type 1 diabetes (upper left slide) showing an islet with peri-insulitis; CD45 brown, synaptophysin red. Remaining slides are from rats three weeks after installation of *E. faecalis* in the pancreatic duct stained for islet hormones and immune cell markers as displayed in each slide. The scale bar represents 100 µm

A detailed morphological description of the inflammation in the pancreas of the 29-year-old organ donor that died at onset of T1D has been reported previously [19]. This donor fulfilled the consensus definition of insulitis [21], and clusters of immune cells were frequently observed close to the islets (insulitis) (Fig. 3). Immunohistochemical staining for CD3 revealed that most cells in the insulitis were T cells. Of these, a majority was CD8^+^, but several CD4+ cells were also observed [19]. The morphological observations in the pancreas of this organ donor adhere well with that reported on subjects with recent onset T1D in the literature [3, 9–11].

#### Rats three weeks after bacterial challenge

The general pancreatic inflammation seen in rats acutely after bacteria translocation was not different when compared to our previous report [19]. This acute inflammation in response to the bacterial challenge was no longer observed three weeks after translocation. Seven of the eight control animals had normal pancreatic architecture with the presence of only occasional immune cells scattered in the pancreatic parenchyma. However, some of the rats showed increased occurrence of fibrosis in the head of the pancreas and one control animal had signs of a small bleeding in the head region of the pancreas with the presence of small numbers of CD68+ , CD20+ , CD3+ and CD4+ and CD3 and CD4+ immune cells. The pancreatic tail region of this animal had very few immune cells and no signs of bleedings.

All animals treated with ductal instillation of a bacterial mixture not including *E. faecalis* showed normal pancreatic architecture with the presence of only occasional immune cells. However, in three of the eight rats injected with *E. faecalis*, some pancreatic lobes in both the head and tail regions contained occasional focal areas of dense accumulations of immune cells (Fig. 3), resembling the organization of lymphatic tissue with the presence of large numbers of T cells (CD3+ , CD8+ and CD4+) and B cells (CD20+). These areas of dense lymphatic tissues seemed randomly distributed in the exocrine pancreas.

The most noticeable finding in these three rats was the presence of insulitis in some lobes of both the head and tail regions of the pancreases. In the affected regions, 20–80% of islets showed insulitis (Fig. 3). Islet architecture remained normal, with the presence of insulin-positive cells preferentially in the center and glucagon-positive cells preferentially in the periphery of the islets (Fig. 3). Phenotypically, a majority of the immune cells in the insulitic lesions were T cells (CD3^+^), but also B cells (CD20^+^) and macrophages (CD68^+^) were common (Fig. 3). CD8+ dominated among the T cells in the insulitic lesions, and many displayed the tissue residence marker CD103 (Fig. 3), but also CD4+ cells were present.

#### Rats six weeks after bacterial challenge

All animals had normal pancreatic architecture with the presence of only occasional immune cells. No rat showed insulitis. However, half of the rats challenged with heat-inactivated bacteria in the ductal system showed slightly increased occurrence of fibrosis in the head of the pancreas.

## Discussion

The intense cellular inflammation triggered acutely after instillation of heat-inactivated bacteria in the ductal compartment [19] seemingly vanished after 3 weeks, and the exocrine pancreas was not different from those of the control rats. Importantly, in about one-third of the animals instilled with heat-inactivated *E. faecalis* and examined after 3 weeks a distinct accumulation of both CD8+ and CD4+ T cells was found within and adjacent to some of the islets (Fig. 3), fulfilling the consensus definition of insulitis [21]. Similar to the human pancreas in Fig. 3 and as frequently reported in humans with T1D [3, 9–12], this insulitis affected a minority of all islets and was observed only in some lobes of the pancreases examined. Notably, also occasional lobes in the splenic part of the pancreas showed distinct insulitis affecting a large proportion of islets, i.e., parts of the pancreas most distal from the intestine and the injection site of the heat-inactivated bacteria.

No deterioration of glucose metabolism was found in rats subjected to an IVGTT (Fig. 1). However, at least two additional circumstances are required if hyperglycemia should develop; (1) repeated lesions that over time would gradually affect increasingly larger volumes of the pancreas and (2) a species unable to regenerate the pancreas between each insult. Rodents able to restore both endocrine and exocrine pancreas even after a 90% pancreatectomy in just a few weeks [26] are therefore not suitable for studying the effects of a single inflammatory insult on long-term glucose metabolism. In spite of the lack of effects on glucose metabolism, instillation of bacteria in the ductal compartment in rats fulfills several criteria of being relevant for human T1D: (1) similar affection of both male and female animals, (2) dependency on environmental trigger(s), (3) remitting and relapsing disease process, (4) patchy affection of the pancreas, (5) initial innate inflammation of some pancreatic lobes, and (6) subsequent formation of insulitis, mainly consisting of T cells, in some of the islets.

A constant and puzzling finding, observed already after a few hours after instillation of heat-inactivated bacteria, was the accumulation of a large number of granulocytes and monocytes in the peri-islet area of some islets in lobes not, or only marginally, affected by the inflammation in the exocrine pancreas [19]. Rats examined 3 weeks after bacterial challenge showed no signs of general discomfort or macroscopic pancreatic abnormalities. This observation indicates that the instillation of heat-inactivated bacteria induced a self-resolving short-term patchy inflammation. However, about one-third of the rats instilled with heat-inactivated *E. faecalis* showed persistent insulitis consisting mainly of CD8+ T cells (Fig. 3). This type of insulitis is seemingly identical to that found in human subjects examined a few weeks after diagnosis of T1D, e.g., in the DiViD biopsy study [11, 14], as well as in the pre-diabetic stage in the NOD mouse model of T1D [5]. Notably, a fraction of the T cells found in the insulitic lesions in the rats expressed the CD103 antigen (Fig. 3). The precise role of CD103 on T cells is not fully understood; however, it has been suggested that CD103 is important for conjugation of CD8^+^ T cell to E-cadherin-expressing epithelial cells, thereby facilitating their destruction upon virus and bacterial infections [17]. Recently, we reported on the presence of significant number of tissue resident memory T cells (CD8+ , CD69+ , CD103+) in the insulitic lesions in humans with recent onset T1D [14] similar to the herein reported expression of CD103 on T cells in the insulitis in rats after triggering innate immunity. Also, the areas of dense lymphatic tissues seemingly randomly distributed in the exocrine pancreas in the rats (Fig. 3) mimic the compartmentalized tertiary lymphoid organs recently reported in human subjects with short T1D disease duration [27, 28]. Similarly, the rats showed small multifocal infiltrates of CD3+ cells in acinar regions (Fig. 3) identical to those described in subjects with recent onset T1D [9].

A role for bacteria in the development of T1D is emerging [19, 29–31]. Notably, both the type of and the intensity of the proinflammatory responses induced in isolated human islets dependent on the specific strain of bacteria applied [19, 31]. Although the present study includes only a few bacterial species, only rats instilled with a bacterial challenge including heat-inactivated *E. faecalis* developed longstanding insulitis. Expression of genes related to anti-bacterial response was upregulated in the pancreas of rats exposed to heat-inactivated bacteria relative to that observed in controls and similar to that found in the subject with recent onset T1D (Fig. 2). It is therefore postulated that the observed findings result as an interplay between an external inflammatory trigger (the heat-inactivated bacteria) and a proinflammatory response induced in affected islets (release of cytokines and chemokines) resulting in the formation of insulitis.

Trafficking and activation of leukocytes is controlled by chemokines and cytokines produced by parenchymal cells in response to inflammation. The HLA genotypes conferring an increased risk for T1D are linked to innate responses to bacterial infections [32] further underlying the importance of the interplay between innate and acquired immune responses in T1D. Several proteins with this powerful immunoregulatory capacity are produced by human islet cells, e.g., CCL2 (MCP-1), CCL5 (RANTES), CCL3 (macrophage inflammatory protein 1-alpha (MIP-1-alpha)), CXCL2 (MIP-2), CXCL9 (monokine induced by gamma interferon (MIG)), CXCL10 (Interferon gamma-induced protein 10 (IP-10)), CXCL11 (Interferon-inducible T-cell alpha chemoattractant (I-TAC)), Macrophage migration inhibitory factor (MIF), IL-1β, IL-6 and IL-8 [33–35]. This cascade of cytokines and chemokines released by islet cells in response to inflammation would initially recruit mainly granulocytes and monocytes to the islets [19] that, as described herein, at later stages are replaced by T and a few B lymphocytes to form the archetypical insulitis observed in subjects with recent onset T1D [20]. Whenever repeated, similar processes would be initiated in additional lobes of the pancreas eventually affecting large volumes of the pancreas. Tentatively, repeated pancreatic inflammations would induce activation of T_RM_ cells present in preexisting insulitis resulting in beta cell cytolysis until the total number of insulin producing cells is too low to maintain glucose metabolism.

In summary, a previously unknown association between innate immunity and the formation of insulitis, the morphological hallmark of T1D, is described. Further studies are required to unravel the detailed mechanistic interplay between the innate immunity and the formation of several immunopathological events seemingly identical to those described in humans with recent onset T1D.

## Decalarations
